# Association between maternal antimony exposure and offspring congenital heart defects: findings from a large birth cohort study

**DOI:** 10.3389/fpubh.2026.1957018

**Published:** 2026-10-19

**Authors:** Yawen Shao, Lei Cao, Ru Lin, Jie Ran, Qing Liu, Baohong Mao

**Affiliations:** 1 Cervical Cancer Prevention and Treatment Center, Gansu Provincial Maternity and Child-Care Hospital (Gansu Provincial Central Hospital), Lanzhou, Gansu, China; 2 Department of Medical Education, Gansu Provincial Maternity and Child-Care Hospital (Gansu Provincial Central Hospital), Lanzhou, Gansu, China; 3 School of Public Health, Gansu University of Chinese Medicine, Lanzhou, Gansu, China; 4 Department of Obstetrics and Gynecology, Gansu Provincial Maternity and Child-Care Hospital (Gansu Provincial Central Hospital), Lanzhou, Gansu, China

**Keywords:** antimony, congenital heart defects, environmental pollutant, interaction, pregnancy

## Abstract

**Background:**

This nested case–control study aimed to investigate the association between maternal blood antimony concentrations and the risk of congenital heart defects (CHDs) in offspring, identify potential risk factors for CHDs, and inform prevention strategies.

**Method:**

The study was conducted within a birth cohort established jointly by Gansu Provincial Maternity and Child Care Hospital and Yale University. A total of 291 women were enrolled, including 97 women who gave birth to infants with CHDs (case group) and 194 controls. Controls were selected from the same cohort using a 1:2 matching ratio and matched to cases by age and community. Maternal whole blood samples were collected during prenatal hospitalization before delivery, and antimony concentrations were measured by inductively coupled plasma mass spectrometry (ICP-MS). Receiver operating characteristic (ROC) curve analysis was used to determine an antimony exposure cutoff and classify participants into high and low exposure groups. Conditional multivariable logistic regression was performed to assess the association between maternal blood antimony concentrations and the risk of CHDs in offspring.

**Results:**

The mean maternal blood antimony concentration was 13.05 μg/L in the control group and 20.30 μg/L in the CHDs group, and the difference between the two groups was statistically significant. Compared with the low exposure group, the high exposure group was associated with a significantly higher risk of CHDs in offspring (OR = 4.455, 95% CI: 2.029–9.782). Similar associations were observed for the subgroups of isolated CHDs, great arteries malformation, septal defects, and atrial septal defects (ASDs). For each 6 μg/L increment in maternal blood antimony, a 38.9% higher risk of CHDs in offspring (OR = 1.389, 95% CI: 1.110–1.735), with consistent findings in the isolated CHDs, septal defects, and ASDs subgroups. Restricted cubic spline (RCS) analysis showed that when maternal blood antimony exceeded 18.409 μg/L, CHDs risk in offspring increased with rising antimony levels.

**Conclusion:**

Higher maternal blood antimony concentrations were associated with an increased risk of CHDs in offspring. These findings suggest that the prenatal living environment may be a modifiable target for CHD prevention and warrant efforts to minimize exposure to potential environmental sources of antimony.

## 1 Introduction

Congenital heart defects (CHDs) are defined as structural abnormalities of the heart and great vessels that are present at birth (1). The global prevalence of CHDs is approximately 8 to 10‰, with substantial variation across geographical regions. Among these regions, Africa had the lowest prevalence (approximately 2.315‰), whereas Asia had the highest (approximately 9.342‰) (2). CHDs are the most common type of congenital malformation and a leading cause of perinatal mortality (3), representing a significant public health issue and imposing a considered social burden. The etiology of CHDs is considered multifactorial, resulting from the interplay of genetic and environmental factors during embryonic development (4, 5).

With industrial development and changes in the living environment, growing attention has been directed to the association between prenatal environmental factors (such as industrial pollutants, heavy metal exposure, and occupational hazards) and adverse pregnancy outcomes (6–8). Studies have suggested that heavy metal exposure during pregnancy may be a risk factor for CHDs (9, 10). Due to regional differences in living environments, the prevalence and distribution of types of CHDs vary across regions (11). Therefore, identifying the major risk factors for CHDs in specific regions is of considerable clinical importance for the prevention and management of CHDs.

Antimony is a toxic trace element and a globally concerning environmental pollutant. It is widely applied in the production of flame retardants, automotive brake friction materials, and lubricants (12). Additionally, antimony is abundant in airborne particulate matter (13, 14). Antimony can accumulate in human and animal bodies via inhalation, dietary intake, and dermal contact. Existing studies have indicated that antimony exposure is associated with vascular inflammation, oxidative stress, and hypertension (15). However, epidemiological evidence regarding the association between antimony exposure and CHDs remains scarce. Accordingly, based on an established birth cohort, the present nested case–control study investigated the association between maternal blood antimony concentrations and CHDs in newborns, aiming to provide empirical evidence for the targeted prevention of CHDs.

## 2 Materials and methods

### 2.1 Study population

The study population was recruited from a birth cohort study established by Gansu Provincial Maternity and Child-care Hospital in Lanzhou, China (7, 16). All participants signed informed consent and completed standardized one-on-one, face-to-face questionnaires administered by trained investigators at enrollment, with follow-up continuing until delivery. Both case and control groups were enrolled from this cohort. Detailed recruitment procedures have been reported in our previous study (17). The case group consisted of 97 mother–newborn pairs. The control group included 194 mother–newborn pairs, which were matched to cases at a 1:2 ratio based on maternal age (within 2 years) and residential community. All controls participants delivered singleton, full-term, newborns with normal birth weights and no congenital malformations. The final study population comprised 291 comprised, including 97 cases and 194 controls.

### 2.2 Questionnaire interview

Following informed consent acquisition, uniformly trained investigators conducted one-on-one face-to-face interviews to administer standardized questionnaires. The main content of the questionnaire included maternal age, ethnicity, employment status, place of residence, education level, smoking during pregnancy, pregnancy history, childbirth history, presence of gestational hypertension or anemia, folic acid supplementation during pregnancy, neonatal gender, and gestational week at delivery. Relevant delivery information was extracted and recorded from official hospital delivery records.

### 2.3 Classification of congenital heart defects

Neonatal cardiac screening and ultrasonic examination were performed within 3 days postpartum to identify abnormal cardiac development. A follow-up ultrasound examination at 42 days after delivery was further conducted to confirm the final CHDs diagnosis. All CHDs cases were classified and coded according to the ICD-10 diagnostic criteria (Q20–Q28), as follow: (1) Great arteries malformations (Q25): including patent ductus arteriosus (PDA) (Q25.0), aortic stenosis, pulmonary stenosis, pulmonary atresia, and other great arteries malformations. (2) Septal defects (Q21): including atrial septal defects (ASDs) (Q21.1), ventricular septal defects, endocardial cushion defect, single atrium, and other cardiac septum malformations. (3) Other categories: congenital malformations of cardiac chambers and connections, congenital malformations of the aortic and mitral valves, and other structural malformations of the heart. The diagnosis criteria of complex congenital heart defects (CCHDs) were defined according to standards established by the U. S. Centers for Disease Control and Prevention (CDC) (18, 19), covering aortic stenosis, double outlet right ventricle, complete transposition of the great arteries, hypoplastic left heart syndrome, interrupted aortic arch, pulmonary atresia, Tetralogy of Fallot, and tricuspid atresia.

### 2.4 Covariates

The study covariates were categorized as follows: (1) Socio-demographic characteristics, including maternal age (<30 and ≥30), maternal nation (Han and Minority), maternal education level (high school or below, college, master’s degree or above), body mass index (BMI) during pre-pregnancy (underweight, normal weight, overweight, and obesity), gestational weight gain (<15, 15–18.5, ≥24), preconception health education (No/Yes), maternal employment (No/Yes), monthly income (≤2,000, 2,000–4,000, ≥ 4,000 RMB per capita), and smoking during pregnancy (passive and active), alcohol consumption during pregnancy. Passive smoking was defined as exposure to another person’s tobacco smoke for more than 15 min/day. (2) Maternal health-related factors including Folic acid supplement (whether the duration of supplementation was ≥12 weeks), Vitamin or mineral supplement (whether the duration of supplementation was ≥ 12 weeks), gestational hypertension (No/Yes), and neonatal sex.

### 2.5 Blood collection, storage and analysis

Maternal blood samples were collected from all participants during prenatal hospitalization. Specifically, 5 mL of fasting peripheral blood was obtained from each pregnant woman in the early morning and stored in an ultra-low temperature freezer at −80 °C. All sample-related information was systematically recorded in the electronic biospecimen database. Frozen blood samples were thawed at room temperature, and 0.2 mL of each sample was transferred into digestion vessels. Subsequently, 1 mL of 68% HNO_3_ and 0.2 mL of 40% H_2_O_2_ were added successively. The samples were then placed in an oven and digested at 175 °C for 6–8 h. After cooling to room temperature, the resulting digestate was transferred into a 10 mL volumetric flask and diluted to the calibration mark with deionized water. The solution was thoroughly homogenized prior to instrumental analysis.

Maternal blood antimony (Sb) concentrations were measured by inductively coupled plasma mass spectrometry (ICP-MS, model ICAP RQ, Thermo Fisher, United States) (20). The operating parameters for ICP-MS were set as follows: peristaltic pump speed at 40.0 rpm; auxiliary gas (high-purity argon, purity > 99.999%) flow rate at 0.8 L/min; nebulizer gas (argon) flow rate at 1.0 L/min; cooling gas (argon) flow rate at 14.0 L/min. The sampling cone aperture was 1.1 mm, and the skimmer cone diameter was 0.75 mm. High-purity helium was applied as the collision gas at a flow rate of 4.1 mL/min. Peak-jumping mode was adopted for scanning, with an RF power of 1,400 W, dwell time of 10 ms, sampling depth of 12 mm, and three replicate scans for each sample.

Each ICP-MS instrument calibration had to satisfy the following performance criteria: under standard mode (STD), the signal intensities of ^115^In should exceed 2.2 × 10^5^, ^7^Li > 5.5 × 10^4^, ^59^Co > 1.0 × 10^5^, and ^238^U > 3.3 × 10^5^; under kinetic energy discrimination mode (KED), the signal intensity of ^59^Co should exceed 3.0 × 10^4^, the Ce oxide ratio (155/140) should be less than 0.02, and the Co/ClO ratio should be greater than 18. Digestion reagent blanks and instrument blanks were included in each analytical batch. Mixed internal standards (^103^Rh, ^45^Sc, ^72^Ge, ^115^In, ^150^Re and ^7^Li) were applied for on-line correction to compensate for blood matrix effects and instrumental signal drift. The internal standard solution was a multi-element reference standard (33 elements, 1,000 μg/mL) obtained from SPEX CertiPrep. Reagents for quality control, calibration, and sample testing were all platform-supplied accessory reagents for the ICP-MS instrument.

Antimony (^121^Sb) in whole-blood samples was determined by microwave digestion and ICP-MS. The calibration range for antimony was 0–100 μg/L with a correlation coefficient *R*^2^ of 0.9999, and *R*^2^ > 0.9998 was obtained for all target analytes. The method limit of detection (LOD) and limit of quantification (LOQ) for antimony were 0.008 μg/L and 0.020 μg/L, respectively. The method precision for antimony was 3.449%, and the precision for all analytes ranged from 0.142 to 12.856%. The spike-recovery of antimony was 98.36%, and spike-recoveries for all analytes ranged from 73.06 to 106.78%. Laboratory staff performing sample pretreatment and ICP-MS measurements were fully blinded to the case–control status of specimens to minimize potential assessment bias.

### 2.6 Statistical analysis

Data were double-entered in EpiData 3.2, and all statistical analyses were conducted using SAS 9.4. The chi-square test or Fisher’s exact test was applied to compare baseline characteristics between the CHDs group and the control group. Between-group differences in continuous variables were assessed using the independent-samples *t*-test. The Wilcoxon rank-sum test was employed for variables with non-normal distribution. Maternal blood antimony concentrations were summarized using quartiles to describe their distribution. The cutoff value for maternal blood antimony concentration was determined through receiver operating characteristic (ROC) curve analysis. We set maternal blood antimony concentration as the test variable and offspring CHDs occurrence as the status variable, calculated the Youden index for all candidate cut points, and selected the concentration corresponding to the maximum Youden index (Youden index = sensitivity + specificity − 1) as the optimal cutoff value. According to this standard, maternal antimony levels were stratified into high exposure and low exposure groups to evaluate the concentration dependent risk of maternal antimony exposure on offspring CHDs.

Conditional logistic regression models were applied to fit the 1:2 matched nested case–control design. Each matched set consisting of one case and two controls was treated as a separate stratum. Matching factors including maternal age and residential community were controlled through stratification and were not entered as covariates in regression models. The covariates retained uniformly in the final adjusted models included maternal nation, pre-pregnancy BMI, weight gain during pregnancy, smoking (passive and active), gestational hypertension, folic acid supplement, and vitamin or mineral supplement. Conditional multivariate logistic regression was performed to estimate odds ratios (*ORs*) and 95% confidence intervals (*CIs*) for relevant risk factors. We further assessed the interaction between maternal blood antimony concentrations and family income in relation to CHDs risk among offspring. The multiplicative interaction parameters [OR = OR11/(OR01 × OR10)] and 95% CI were also estimated by including the abovementioned variables. The interactions on the additive scale were assessed using relative excess risk due to interaction (RERI = RR11 − RR10 − RR01 + 1), attributable proportion (AP = RERI/RR11), and synergy index [S = (RR11–1)/[(RR01–1) + (RR10–1) (21–24). We estimated 95% CI for each of these measures; the null values of RERI and AP were 0, whereas the null value for S was 1.

Restricted cubic spline (RCS) curves were used to further explore the nonlinear relationship between maternal blood antimony levels and the risk of offspring CHDs. The number of knots was set to three based on comparison of the Bayesian information criterion and Akaike information criterion (25).

Analyses were conducted using SAS, version 9.4 (SAS Institute, Inc., Cary, NC, USA). The RCS models were fitted using R software, version 4.4.2 (packages “Hmisc”, “rms”, and “survival”). The forest plots were generated using R software, version 4.4.2 (packages “grid”, “readxl”, and “forestploter”). All statistical tests were two-sided, and statistical significance was defined at *p*-value < 0.05.

## 3 Results

### 3.1 Basic characteristics of the study population

A total of 291 women were enrolled in this study, including 97 cases who gave birth to infants with CHDs and 194 controls. Baseline characteristics of the study participants are summarized in Table 1. Compared with women in the control group, participants who delivered infants with CHDs had higher odds of gestational hypertension. No significant differences were observed between the two groups with respect to maternal age, maternal ethnicity, maternal education level, pre-pregnancy BMI, gestational weight gain, employment status during pregnancy, household income, smoking status (including passive and active smoking), folic acid supplementation, vitamin or mineral supplementation, or infant sex.

**Table 1:** Distributions of selected characteristics of the study population.

Characteristics	CHDs(97)		Control(194)		*χ* ^2^	*p*-value
	N	%	N	%		
Maternal age					1.438	0.231
<30	56	57.73	126	64.95		
≥30	41	42.27	68	35.05		
Maternal nation					3.520	0.061
Han	90	92.78	189	97.42		
Other minority	7	7.22	5	2.58		
Maternal education level					1.813	0.404
Master’s degree or above	9	9.28	12	6.19		
College	41	42.27	96	49.48		
High school and below	47	48.45	86	44.33		
Pre-pregnancy BMI					3.494	0.174
<18.5	24	24.74	41	21.13		
18.5–24	58	59.79	135	69.59		
≥24	15	15.46	18	9.28		
Weight gain during pregnancy (kg)					3.644	0.162
<15	47	48.45	73	37.63		
15–18.5	18	18.56	51	26.29		
>18.5	32	32.99	70	36.08		
Preconception health education					0.342	0.559
No	52	53.61	111	34.54		
Yes	45	46.89	83	42.78		
Maternal employ					0.188	0.665
No	36	37.11	67	34.54		
Yes	61	62.89	127	65.46		
Per capita monthly income					1.274	0.529
<2,000	31	31.96	53	27.32		
2,000–4,000	36	37.11	85	42.81		
>4,000	30	30.93	56	28.87		
Smoking (passive and active)					0.011	0.918
No	77	79.38	155	79.90		
Yes	20	20.62	39	20.10		
Gestational hypertension					20.712	<0.001
No	87	89.69	194	100.00		
Yes	10	10.31	0	0.00		
Folic acid supplement					1.368	0.242
Supplementary time <12w	27	27.84	42	21.65		
Supplementary time ≥12w	70	72.16	152	78.35		
Vitamin or mineral supplement					0.6152	0.433
Supplementary time <12w	88	90.72	168	86.60		
Supplementary time ≥12w	9	9.28	26	13.40		
Newborn’s sex					0.445	0.505
Male	46	47.42	84	43.30		
Female	51	52.58	110	56.70		

### 3.2 The differences in antimony content in maternal blood among different groups of CHDs

As shown in Table 2, 97 eligible cases were enrolled in this study. Of these, 43 were classified as isolated CHDs (44.3%) and 54 as multiple CHDs (55.7%). The distribution of CHDs subtypes comprised 73 cases of great arteries malformation, 65 cases of PDA, 60 cases of septal defects, and 48 cases of ASDs. The mean maternal blood antimony concentration was 13.05 μg/L in the control group and 20.30 μg/L in the CHDs group, with a statistically significant difference observed between the two groups. Within the isolated CHDs group, the mean maternal blood antimony concentration was 19.50 μg/L, which differed significantly from that of the control group. Nevertheless, no statistically significant differences in maternal blood antimony concentrations were found between the control group and the groups with multiple CHDs, great arteries malformation, PDA, septal defects, or ASDs.

**Table 2:** Comparison of maternal blood antimony levels between the case group and the control group.

Group	Cases	Mean value	P25	P50	P75	*Z*-value	*p*-value
Control	194	13.05	8.80	11.97	16.75	-	-
CHDs	97	20.30	9.02	14.05	20.30	2.061	0.039
The isolated CHDs	43	19.50	10.30	16.84	23.52	2.960	0.003
The multiple CHDs	54	20.93	8.13	13.32	20.30	0.409	0.683
Great arteries malformation	73	20.67	8.62	13.88	20.32	1.470	0.142
PDA	65	21.36	9.05	13.88	20.32	1.524	0.127
Septal defects	60	22.08	9.51	13.93	20.30	1.918	0.055
ASDs	48	16.77	9.51	13.85	20.30	1.675	0.094

### 3.3 The association between maternal blood antimony concentrations and the prevalence of CHDs in offspring

Maternal blood antimony levels were stratified according to the cutoff values (19.994), and multivariate logistic regression analysis was performed to evaluate the association between maternal blood antimony levels and the CHDs risk in offspring, with the low exposure group serving as the reference. As shown in Figure 1, elevated maternal blood antimony levels were associated with increased CHD risk in offspring. Compared with the low exposure group, the high exposure group exhibited a higher overall risk of CHDs in offspring (OR = 4.455, 95% CI: 2.029–9.782), and an association was also observed between high antimony exposure and isolated CHDs (OR = 12.648, 95% CI: 2.809–56.953). However, no significant association was observed for the multiple CHDs subgroup. Separate analyses of CHDs subtypes showed that elevated maternal blood antimony levels were associated with higher CHDs risk in the great arteries malformation, septal defects, and ASDs subgroups. Furthermore, each 6 μg/L increment in maternal blood antimony levels was associated with a 38.9% higher risk of CHDs in offspring (OR = 1.389, 95% CI: 1.110–1.735). Comparable associations were noted in the isolated CHDs, septal defects, and ASDs subgroups.

**Figure 1:**
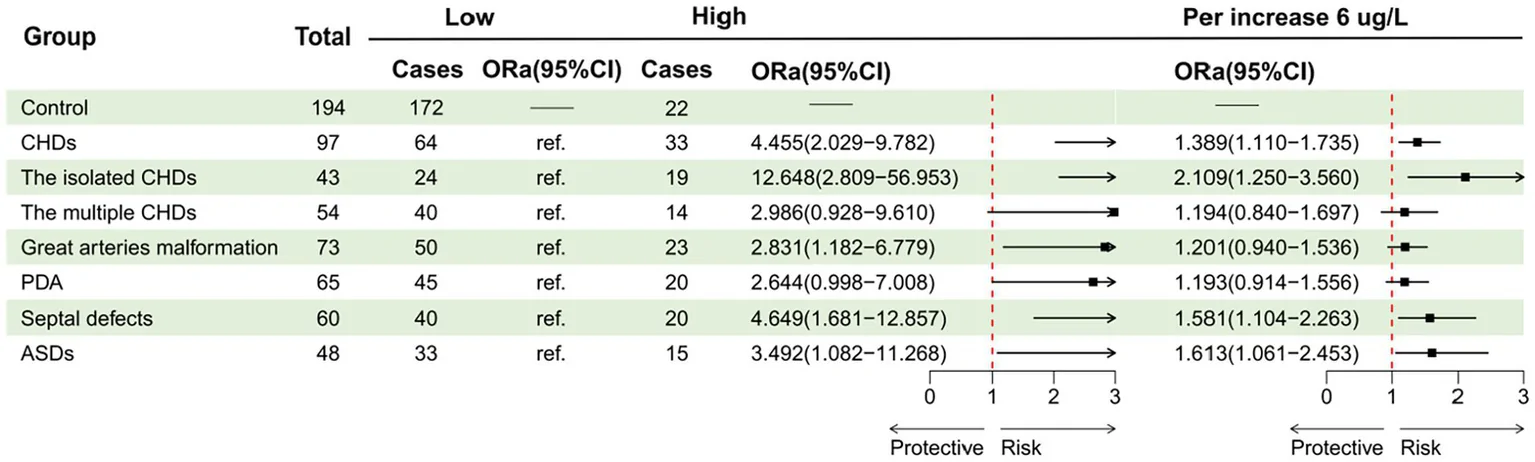
Illustrates the association between maternal blood antimony levels and the risk of CHDs and their subtypes in offspring. ORa was adjusted for maternal nation, pre-pregnancy BMI, weight gain during pregnancy, smoking (passive and active), gestational hypertension, folic acid supplement, and vitamin or mineral supplement.

Figure 2 shows the restricted cubic spline curve illustrating an “inverted L-shaped” association between maternal blood antimony levels and CHDs risk in offspring. At maternal blood antimony levels ranging from 0 to 18.409 μg/L, CHDs risk in offspring remained low and stable. When maternal blood antimony levels exceeded 18.409 μg/L, CHDs risk in offspring increased as antimony concentrations rose.

**Figure 2:**
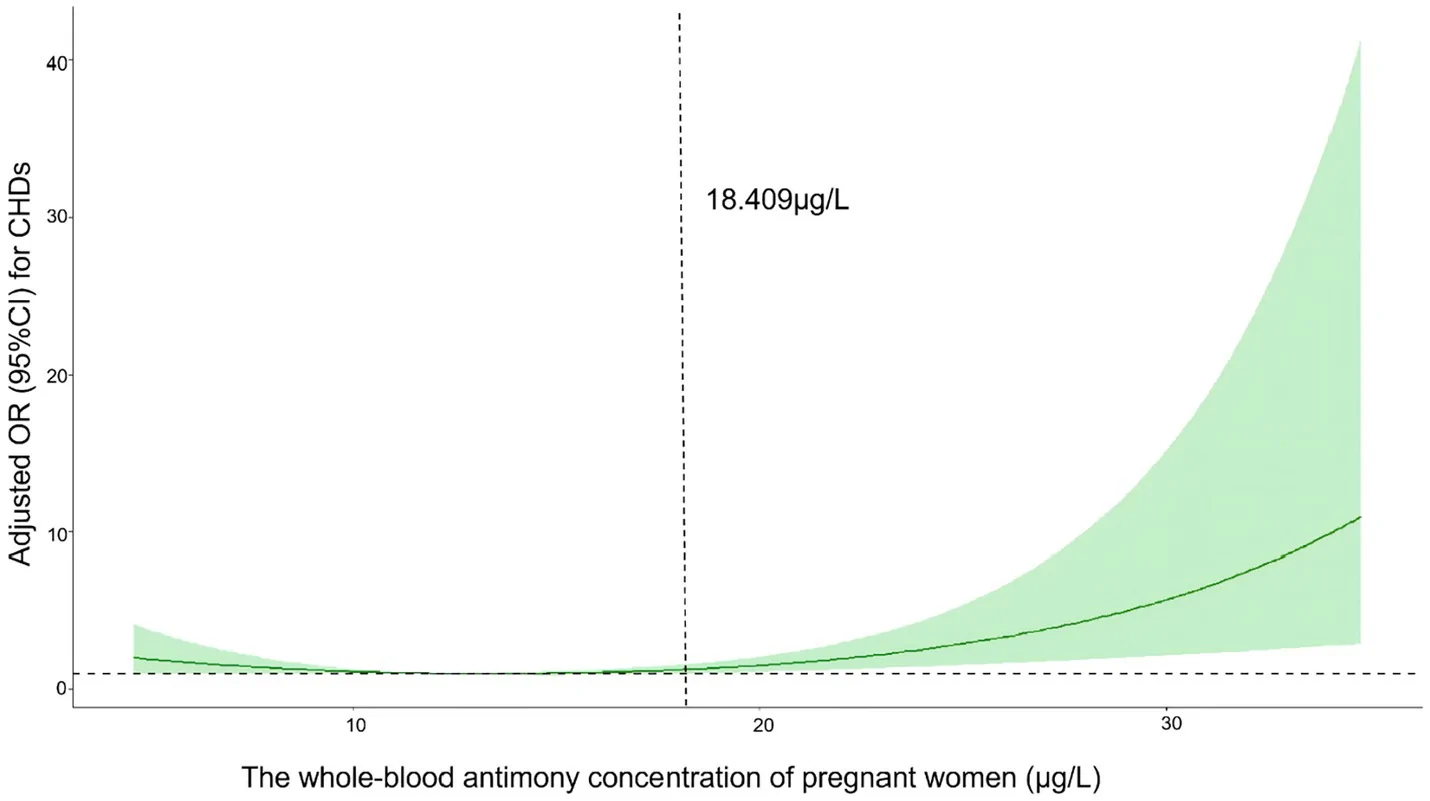
Depicts the restricted cubic spline plot for the association between maternal blood antimony levels and CHDs risk in offspring. The restricted cubic spline (RCS) was adjusted for maternal ethnicity, pre-pregnancy BMI, weight gain during pregnancy, smoking (passive and active), gestational hypertension, folic acid supplementation, and vitamin or mineral supplementation.

### 3.4 Association between maternal blood antimony levels and the risk of CHDs in different subgroups

As shown in the Figure 3, relative to the low exposure group, the high exposure group was associated with significantly higher CHDs risk in both the younger age group (< 30 years) and the older age group (≥30 years). Comparable findings were noted following stratification by weight change during pregnancy, preconception health education, employment status during pregnancy, and timing of folic acid supplementation. After stratification by pre-pregnancy BMI, no significant association was detected between the low and high exposure groups among women with a pre-pregnancy BMI ≥ 24. Subgroup analyses by family income and infant sex showed no statistically significant association for the subgroup with a monthly household income >4,000 or in the male infant subgroup.

**Figure 3:**
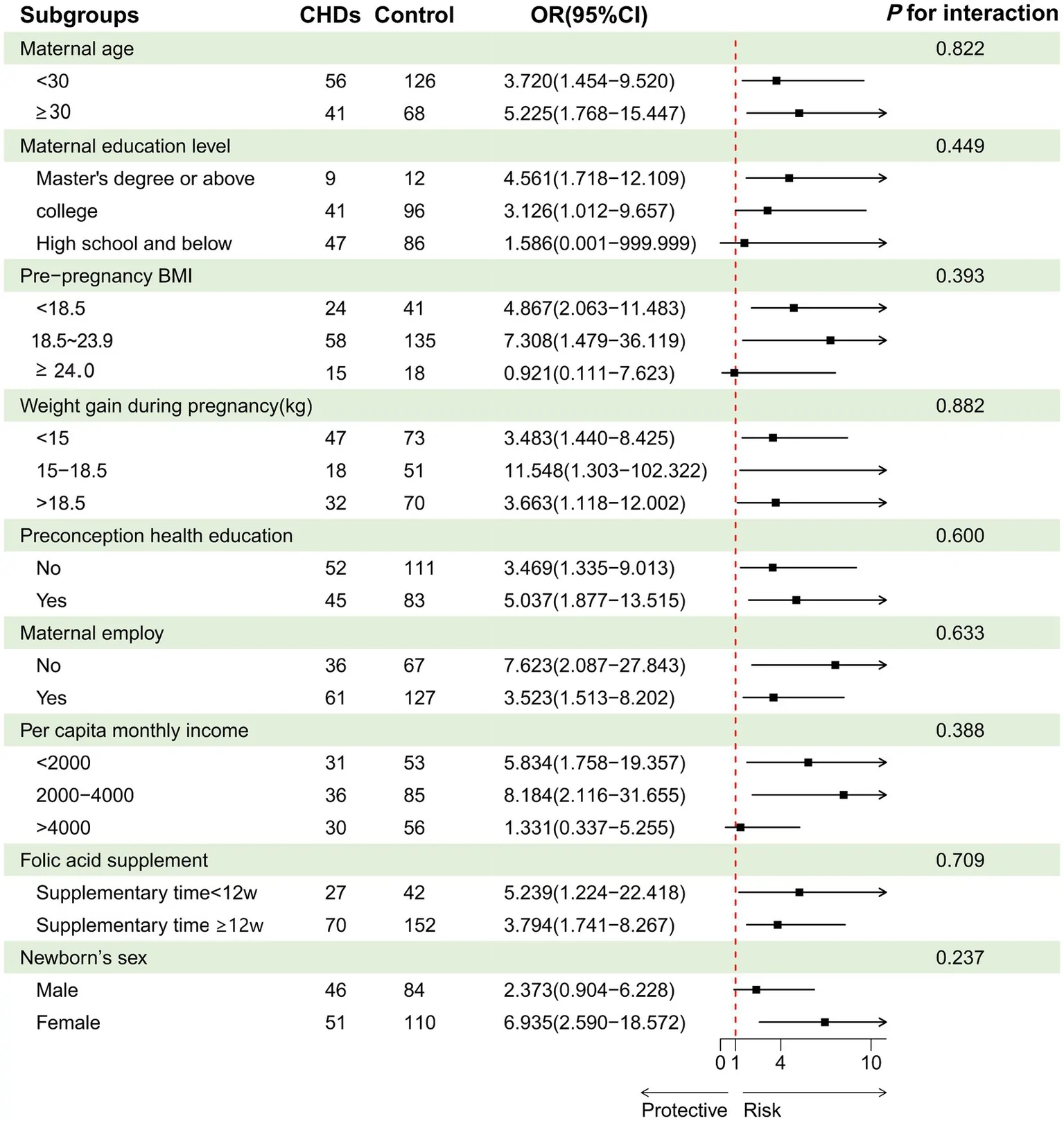
Illustrates the association between maternal blood antimony levels and CHDs risk in offspring across different subgroups. ORa was adjusted for maternal nation, pre-pregnancy BMI, weight gain during pregnancy, smoking (passive and active), gestational hypertension, folic acid supplement, and vitamin or mineral supplement.

### 3.5 Interaction effects of maternal blood antimony and income on the risk of CHDs in offspring

As shown in Table 3, a multiplicative interaction between maternal blood antimony levels and household income with respect to CHDs risk in offspring was identified (OR = 4.477, 95% CI: 2.071–9.681), and the trend test yielded a statistically significant result (*p* < 0.0001). No additive interaction was detected. Consistent associations were observed in subgroups of CHDs, including isolated CHDs, great arteries malformation, PDA, septal defects, and ASDs. Compared with the low blood antimony and high income group, the high blood antimony and low income group was associated with a 65.4% higher risk of CHD in offspring (OR = 1.654, 95% CI: 1.197–2.285). An associations were also noted for isolated CHDs (OR = 2.066, 95% CI: 1.344–3.177), great arteries malformation (OR = 1.457, 95% CI: 1.031–2.060), septal defects (OR = 1.857, 95% CI: 1.239–2.783), and ASDs (OR = 1.645, 95% CI: 1.055–2.565).

**Table 3:** Interaction effects of maternal blood antimony and family income on CHDs in offspring.

Group	CHDs/Control	ORc (95% CI)	ORa (95% CI)
CHDs			
Low antimony and high income	22/47	ref.	ref.
Low antimony and low income	42/125	0.718 (0.388–1.328)	0.774 (0.402–1.492)
High antimony and high income	8/9	1.378 (0.804–2.363)	1.170 (0.602–2.276)
High antimony and low income	25/13	1.602 (1.210–2.119)	1.654 (1.197–2.285)
Multiplicative interaction: ORd (95% CI) = 4.477 (2.071–9.681), *p* < 0.0001			
Additive interaction: RERI (95% CI) = 2.492 (−0.897–5.881), AP (95% CI) = 0.606 (0.075–1.138), S (95% CI) = 5.040 (0.164–154.919)			
The isolated CHDs			
Low antimony and high income	9/47	ref.	ref.
Low antimony and low income	15/125	0.627 (0.257–1.529)	0.633 (0.236–1.696)
High antimony and high income	3/9	1.319 (0.627–2.777)	1.343 (0.580–3.113)
High antimony and low income	16/13	1.859 (1.323–2.614)	2.066 (1.344–3.177)
Multiplicative interaction: ORd (95% CI) = 10.382 (3.995–26.980), *p* < 0.0001			
Additive interaction: RERI (95% CI) = 5.060 (−0.923–11.043), AP (95% CI) = 0.787 (0.382–1.193), S (95% CI) = 14.772 (0.010–22921.518)			
The multiple CHDs			
Low antimony and high income	13/47	ref.	ref.
Low antimony and low income	27/125	0.781 (0.372–1.640)	0.900 (0.409–1.980)
High antimony and high income	5/9	1.417 (0.757–2.653)	0.895 (0.306–2.619)
High antimony and low income	9/13	1.358 (0.957–1.926)	1.322 (0.841–2.080)
Multiplicative interaction: ORd (95% CI) = 1.927 (0.685–5.420), *p* = 0.2138			
Additive interaction: RERI (95% CI) = 0.713 (−2.366–3.793), AP (95% CI) = 0.285 (−0.818–1.388), S (95% CI) = 1.904 (0.067–54.283)			
Great arteries malformation			
Low antimony and high income	19/47	ref.	ref.
Low antimony and low income	31/125	0.613 (0.316–1.189)	0.653 (0.326–1.307)
High antimony and high income	6/9	1.284 (0.718–2.296)	0.963 (0.449–2.064)
High antimony and low income	17/13	1.479 (1.096–1.994)	1.457 (1.031–2.060)
Multiplicative interaction: ORd (95% CI) = 3.477 (1.477–8.186), *p* = 0.0043			
Additive interaction: RERI (95% CI) = 1.972 (−0.975–4.919), AP (95% CI) = 0.610 (−0.014–1.234), S (95% CI) = 8.511 (0.004–17989.259)			
PDA			
Low antimony and high income	16/47	ref.	ref.
Low antimony and low income	29/125	0.681 (0.340–1.367)	0.698 (0.337–1.443)
High antimony and high income	6/9	1.400 (0.776–2.523)	1.030 (0.464–2.290)
High antimony and low income	14/13	1.468 (1.071–2.011)	1.356 (0.921–1.997)
Multiplicative interaction: ORd (95% CI) = 2.601 (1.029–6.570), *p* = 0.0433			
Additive interaction: RERI (95% CI) = 1.524 (−1.632–4.680), AP (95% CI) = 0.482 (−0.294–1.257), S (95% CI) = 3.381 (0.084–136.648)			
Septal defects			
Low antimony and high income	12/47	ref.	ref.
Low antimony and low income	28/125	0.877 (0.412–1.866)	1.047 (0.486–2.253)
High antimony and high income	5/9	1.475 (0.784–2.775)	1.472 (0.579–3.738)
High antimony and low income	15/13	1.653 (1.194–2.289)	1.857 (1.239–2.783)
Multiplicative interaction: ORd (95% CI) = 3.957 (1.651–9.484), *p* = 0.0020			
Additive interaction: RERI (95% CI) = 2.466 (−1.758–6.690), AP (95% CI) = 0.546 (−0.097–1.189), S (95% CI) = 3.341 (0.232–48.050)			
ASDs			
Low antimony and high income	10/47	ref.	ref.
Low antimony and low income	23/125	0.865 (0.383–1.953)	1.000 (0.424–2.356)
High antimony and high income	5/9	1.616 (0.848–3.078)	1.479 (0.581–3.762)
High antimony and low income	10/13	1.535 (1.074–2.193)	1.645 (1.055–2.565)

Multiplicative interaction: ORd (95% CI) = 2.685 (1.000-7.205), *p* = 0.0499 Additive interaction: RERI (95% CI) = 1.139 (-3.007-5.285), AP (95% CI) = 0.315 (-0.679-1.310), S (95% CI) = 1.772 (0.168-18.683). ORc crude odds ratio. ORa was adjusted for maternal nation, pre-pregnancy BMI, weight gain during pregnancy, smoking (passive and active), gestational hypertension, folic acid supplement, and vitamin or mineral supplement.

## 4 Discussion

This nested case–control study comprehensively explored the association between maternal blood antimony concretrations during pregnancy and CHDs risk in offspring, and identified a dose–response relationship between blood antimony levels and CHDs risk in offspring. Furthermore, we found that each 6 μg/L increment in maternal blood antimony levels was associated with an elevated risk of offspring CHDs, and consistent findings were observed in subgroup analyses of common CHDs types. Furthermore, we observed that several CHDs subtypes showed no statistically significant associations in the continuous antimony exposure model, yet significant associations emerged after stratification by the cutoff value. This observation suggests a potential threshold-dependent effect of antimony: elevated risk occurs only above a certain exposure concentration, rather than a linear dose–response relationship across the full exposure range. Meanwhile, limited statistical power due to small case numbers for individual subtypes may also account for this discrepancy. Dichotomized analysis enables outcome comparisons between high and low exposure groups; however, this approach and the continuous linear model test different statistical assumptions. Although we adjusted for potential confounders, residual confounding cannot be excluded. Accordingly, subtype-specific results derived from categorical analyses should be interpreted with caution, and further validation in larger cohort studies is warranted.

CHDs are a common birth defect and a leading cause of death among children under 5 years of age (26), with an incidence ranging from 0.5‰ to 9‰ (11, 27, 28). In China, CHDs rank first among all birth defects, with a prevalence of 6‰ to 8‰ (29). The exact etiology of CHDs remains incompletely understood; current evidence suggests that CHDs arise from combined effects of genetic and environmental factors. Previous studies have demonstrated that adverse psychological factors, alcohol consumption, residential noise exposure, and exposure to harmful substances during early pregnancy increase the risk of CHDs (4, 30). Additionally, traffic-related air pollution and exposure to pollutants released from residential decoration materials have also been associated with CHDs (26). In recent years, environmental heavy metal exposure has attracted increasing attention for its potential association with elevated CHDs risk. Existing studies have indicated that maternal peripheral blood levels of heavy metals, including lead, cadmium, arsenic, and mercury, are significantly higher in mothers of children with CHDs than in the general population (9, 31). Furthermore, considering that the prevalence of CHDs varies by altitude and even across ethnic groups within the same altitude zone, targeted research on region-specific CHDs risk factors is warranted (29). Existing studies have mainly focused on the eastern coastal areas of China, while evidence from northwestern regions remains limited. Furthermore, few studys have explored the association between antimony, a priority-controlled pollutant, and CHDs risk. Accordingly, we performed the present study among permanent residents in an urban area of northwestern China. Residents from mining zones and other sites with elevated antimony levels were excluded to reduce selection bias, so that the findings could be reasonably representative.

Antimony is a non-essential trace element for humans and exhibits chemical properties similar to arsenic. It is ubiquitous in modern human environments; sources including fossil fuels and waste conbustion, computer-chip manufacturing, and electronic-waste recycling may contribute to antimony release into the environment (32). Additionally, plastic containers for mineral-water storage may also contain considerable amounts of antimony (33). Antimony can enter the human body via inhalation, dietary intake, or dermal contact (34). Studies have indicated that long-term exposure to high concentrations of antimony compounds may increase disease risk by disrupting multiple various cellular signaling pathways (35).

A case–control study conducted in China reported that elevated antimony concentrations in the maternal hair represented a risk factor for CHDs (36). One cross-sectional study identified a significant positive dose–response relationship between eight urinary heavy metals (manganese, iron, copper, zinc, cadmium, antimony, barium and lead) and urinary 8-iso-prostaglandin F2α (8-iso-PGF2α), a biomarker of lipid peroxidation (37). Investigators also reported that certain urinary heavy metals (manganese, iron, cadmium, and antimony) were negatively correlated with one or more heart-rate-variability parameters. In an animal experiment, exposure to high-concentration antimony (V) as L-antimony potassium tartrate (purity: 99.5%) significantly altered heart rate and induced developmental delay in zebrafish larvae (38, 39). Furthermore, antimony (V) exposure increased reactive oxygen species (ROS) levels in zebrafish larvae, accompanied by enhanced antioxidant enzyme activity and glutathione (GSH) depletion (35). Quantitative gene expression analysis confirmed that high concentrations of antimony (V) altered the expression of immune/defense response genes in larvae, including stimulator of interferon genes (sting1), interferon regulatory factor 1a/1b (irf1a/irf1b), immunoresponsive gene 1 like (irg1l), and toll-like receptor 22 (tlr22), thereby potentially disrupting immune homeostasis. These findings indicate that antimony (V) induced oxidative stress and immune dysfunction may serve as key mechanisms underlying the developmental toxicity of antimony in zebrafish. Another animal experiment reached consistent conclusions, showing that potassium antimony tartrate (PAT; CAS: 28300-74-5; purity: 99.9%) induces developmental toxicity and neurotoxicity in zebrafish embryos by modulating the neurotransmitter system and oxidative stress (40). Following antimony exposure, zebrafish larvae presented elevated ROS levels and malondialdehyde (MDA) content, accompanied by marked reductions in superoxide dismutase (SOD) activity and GSH content. The larvae also showed abnormal phenotypes, including shortened body length, morphological deformities, and impaired cardiac function. Animal experiment have demonstrated that arsenic (III) (4 mg/kg As_2_O_3_) and antimony (III) (15 mg/kg SbCl_3_) trigger endoplasmic reticulum (ER) stress by disrupting calcium homeostasis (41). Such ER stress further triggers apoptosis in mouse cardiomyocytes by activating the CHOP, JNK, and caspase-12 signaling pathways. Moreover, noticeable mitochondrial swelling and cristae fracture were observed, indicating the occurrence of mitochondrial damage. Cellular experiments have shown that antimony (III) induces oxidative stress and cytotoxicity in mouse cardiomyocytes by disrupting the Ca signaling pathway (42). Consistent cellular experimental findings further demonstrated that both antimony (V) and antimony (III) suppress ATP production and elevate ROS (43). These alterations promote excessive phosphorylation and oxidative stress, ultimately impairing mitochondrial function in isolated mouse cardiac mitochondria. Collective evidence from population-based epidemiological studies, as well as animal and cellular experiments, indicates that antimony exposure may induce toxic demage to the cardiovascular and nervous systems via oxidative stress, mitochondrial dysfunction, and endoplasmic reticulum stress, ultimately increasing the risk of CHDs and other adverse health outcomes.

The potential mechanisms underlying the association between antimony exposure and elevated CHDs risk are summarized as follows. First, antimony can irreversibly bind to intracellular sulfhydryl groups, thereby impairing the normal function of sulfhydryl-containing proteins and enzymes (44, 45). Second, antimony may disrupt the regulatory pathways of cell growth and embryonic development by affecting DNA methylation of specific genes (46, 47). In addition, antimony exposure can trigger systemic oxidative stress, and excessive oxidative stress further causes DNA oxidative damage and lipid peroxidation (48, 49). Although hypothesized mechanisms regarding sulfhydryl binding, DNA methylation, and oxidative stress are biologically plausible, they remain speculative. As an observational study, our work only provides epidemiological clues and cannot establish causal relationships. Further experimental research is required to clarify relevant biological pathways. Glutathione co incubation assays combined with ICP-MS can explore interactions between antimony and sulfhydryl containing molecules. Targeted methylation analyses may screen epigenetic changes in key genes related to cardiac development. Detection of oxidative stress biomarkers alongside antioxidant intervention tests can be conducted using cardiomyocyte models or animal models with gestational antimony exposure to verify these mechanistic hypotheses.

This study showed that maternal peripheral blood antimony levels were higher in mothers of children with CHDs compared to mothers who delivered healthy newborns. Among isolated CHDs, elevated maternal blood antimony concentrations were associated with a higher risk of CHDs. Further analysis revealed that for each 6 μg/L increment in maternal blood antimony levels was associated with higher odds of common CHDs subtypes, including great arteries malformation, PDA, septal defects, and ASDs. These findings are consistent with those reported in previous studies. The in drinking-water antimony limit is 5 μg/L in China and 6 μg/L in the United States (50, 51). We examined CHD risk in relation to antimony levels above the Chinese reference standard, and the results showed that each 6 μg/L increase in antimony concentration was associated with higher odds of isolated CHDs as well as each of the common CHDs subtypes.

As the most common congenital malformation, CHDs impose substantial psychological and economic burdens on affected families and society, representing a key priority for birth defect prevention and control efforts in China. In-depth exploration of the etiology and pathogenesis of CHDs is essential for the prevention and clinical management of this disease. This study suggests that prenatal environmental heavy metal exposure may serve as a risk factor for CHDs, providing a scientific basis for CHDs prevention. Raising awareness regarding maternal living-environment risks, improving self-health consciousness, adopting healthy lifestyles, and avoiding exposure to risk factors may represent potential measures to lower CHDs risk.

### 4.1 Strengths and limitations

Our study used maternal whole-blood antimony as the exposure biomarker to examine the associations between antimony exposure and CHDs and their common subtypes, and systematically evaluated the dose–response relationship between maternal antimony exposure and the risk of offspring CHDs. A nested case–control design was employed, with both cases and controls derived from the same birth cohort and matched at a 1:2 ratio. This approach helped minimize selection bias, enhance statistical efficiency despite the relatively small sample size, and ensure the overall quality of the study. However, certain limitations should be acknowledged. As an observational study, causal inference cannot be drawn from the present analysis. In addition, maternal blood samples were collected before delivery, whereas the critical window for fetal cardiac morphogenesis occurs at gestational weeks 3.5–8. Antimony levels measured prior to delivery may not precisely reflect specific exposure during this critical window, which may lead to potential exposure misclassification. Given the relative stability of maternal living environments during pregnancy together with the accumulation property of antimony in human bodies, the detected concentrations may represent cumulative gestational exposure rather than exposure specific to this critical window. Nevertheless, longitudinal repeated measurements of blood antimony across gestation are lacking in our cohort, so the temporal stability of blood antimony during pregnancy remains unconfirmed, and pre-delivery antimony cannot serve as a reliable proxy for exposure during the critical window. Notably, although structural cardiac malformations mainly originate within this critical window, antimony exposure may still affect subsequent cardiac maturation during late pregnancy and after birth. Furthermore, the total number of CHDs cases in this study was limited, leading to limited statistical precision. Larger-sample multicenter prospective cohort studies are needed to validate our findings, with maternal biological samples collected across different gestational periods to more precisely evaluate the association between antimony exposure during the critical window and cardiac development.

## 5 Conclusion

This study indicates that elevated maternal blood antimony levels may be associated with an increased risk of CHDs in offspring. Therefore, attention to the living environment during pregnancy and avoidance of relevant risk-factor exposure may represent potential measures to lower CHDs risk.

## Data Availability

The datasets generated and/or analyzed during the current study are not made public as they contain private information. However, they can be obtained from the corresponding author upon reasonable request. Requests to access the datasets should be directed to Baohong Mao, Email: Baohong.Mao@gszy.edu.cn.
